# 

**Published:** 1883-11-20

**Authors:** 


☞ RECEIPTS for subscriptions will appear in our next issue.

### *PAMPHLETS RECEIVED.*

American Business Directory,—Containing the names of manufacturers, wholesale merchants, banks, bankers, etc., in the following named cities. to-wit: Baltimore, Boston, Brooklyn, Chicago, Cincinnati, Cleveland, Ohio; Detroit, Michigan; Kansas City, Milwaukee, Wis.; New York, Philadelphia, Pittsburg; Pa.; St Louis, Mo. Published monthly by the American Business Directory Company. New York, 132 Nassau Street; Chicago, 133 La Salle Street.

Closing Exercises of the Practitioners' Course of Lectures, delivered in the spring of 1883, in the Hahnemann Medical College and Hospital of Chicago, Illinois.

A Catalogue of Medical and Surgical books, periodicals and transactions from the Libraries of two physicians, lately deceased, which are offered at very low prices, by Robert Clarke & Co., 61, 63 and 65 West Fourth Street, Cincinnati, Ohio.

Lecture XIV, at the College for Medical Practitioners, St. Louis. Diagnosis of Ovarian Tumors, by Edward Borck, M. D.. 225 Washington Avenue, St. Louis, Missouri.

REMARKS ON HYDROPHOBIA—By Charles W. Dulles, M. D. Reprinted from the Philadelphia Medical Times of August, 1883.

ANNUAL REPORT of the Board of Regents of the Smithsonian Institute, showing the operations, expenditures and condition of the Institution for the year 1881.—Washington Government office, 1883. A Scientific publication containing many interesting facts and observations.

